# Maintenance eculizumab dose adjustment in the treatment of atypical hemolytic uremic syndrome: a case report and review of the literature

**DOI:** 10.1002/ccr3.628

**Published:** 2016-07-06

**Authors:** Nick Thomson, Matthew Ulrickson

**Affiliations:** ^1^ Department of Internal MedicineUniversity of Arizona College of Medicine PhoenixPhoenixArizonaUSA; ^2^ Department of Hematology/OncologyBanner MD Anderson Cancer CenterGilbertArizonaUSA

**Keywords:** Atypical hemolytic uremic syndrome, eculizumab, thrombotic microangiopathies

## Abstract

Atypical hemolytic uremic syndrome (aHUS) patients treated with eculizumab may require higher doses to achieve and maintain optimal clinical response. Further studies are warranted to elucidate optimal dosing regimens of eculizumab in aHUS patients, and whether dosing regimens can be predicted based on mutational status, eculizumab levels, or other testing.

## Introduction

Atypical hemolytic uremic syndrome (aHUS) is caused by a disorder in complement regulation, which leads to leukocyte and platelet activation, thrombotic microangiopathy, and subsequent end‐organ damage 1. It is distinguished from typical hemolytic uremic syndrome (HUS) in that it lacks coexisting disease such as Shiga‐toxin‐producing *Escherichia coli* (STEC) and *Streptococcus pneumoniae* 2. Common causes of atypical HUS include complement gene mutations, which the majority of patients who suffer from aHUS carry, while antibodies to complement factor H have also been implicated in a smaller subset of patients 3, 4. Classic laboratory findings include hemolytic anemia, thrombocytopenia, and acute kidney injury 5. Atypical HUS is distinguished from thrombotic thrombocytopenic purpura (TTP) by its lack of reduction in activity of ADAMTS13. Untreated aHUS is associated with permanent renal dysfunction, high recurrence rate, and high mortality 6. Historically, prognosis was poor: studies showed that 33–40% of patients progressed to ESRD or death during the first clinical manifestation 3. Within 1 year of diagnosis, up to 65% of patients managed with plasma exchange (PEX) suffered permanent renal damage, progressed to ESRD, or died 7. The elucidation of the pathogenesis from uncontrolled activation of the alternative complement pathway, resulting in formation of the membrane attack complex (MAC) C5b‐9 8 has ultimately led to treatment with eculizumab, a monoclonal IgG antibody that binds to C5 and prevents subsequent formation of terminal complement 9. Benefit from eculizumab was initially demonstrated in patients with paroxysmal nocturnal hemoglobinuria (PNH) 10 and subsequently in the treatment of aHUS 11, 12. Dosing of eculizumab in the treatment of aHUS is 900 mg IV weekly for induction for 4 weeks followed by maintenance at 1200 mg IV every 2 weeks 13. These recommended doses were extrapolated from the data in PNH, as the frequency of aHUS did not allow more specific investigation prior to approval of the agent. Dosing that adequately controls complement activation is necessary to achieve optimal outcomes for patients.

## Case Presentation

A 26‐year‐old previously healthy Hispanic male presented with 2 days of epigastric abdominal pain with associated nausea, vomiting, and gross hematuria. He was found to have acute kidney injury (Cr 4.07 mg/dL), thrombocytopenia (PLTs 70K/mm^3^), and microangiopathic hemolytic anemia as evidenced by schistocytes seen on a peripheral blood smear. Treatment for presumed TTP was initiated at an outside institution with high‐dose intravenous steroids and plasmapheresis. In spite of this treatment, his renal function continued to decline with creatinine increasing to a peak of 9 mg/dL. Hemodialysis was initiated on day 4 of presentation. His ADAMTS13 activity was found to be within normal limits (124%). After transfer, quantitative assessment of complement revealed levels within normal limits, includingC3 and C4 at 97 and 39 mg/dL, respectively.

Pneumococcal HUS, HIV, and drug‐related thrombotic microangiopathy were excluded in our patient's workup. Subsequent quantitative complement assessment did reveal abnormally low levels, including C3 and Complement Factor B of 68 and 16 mg/dL, respectively. The patient was found to have very low CH50 of <10 units/mL. A diagnosis of aHUS was made, plasmapheresis was discontinued, and eculizumab therapy was initiated shortly after immunization against *N. Meningitidis*. Ceftriaxone prophylaxis was administered for 2 weeks.

**Figure 1:**
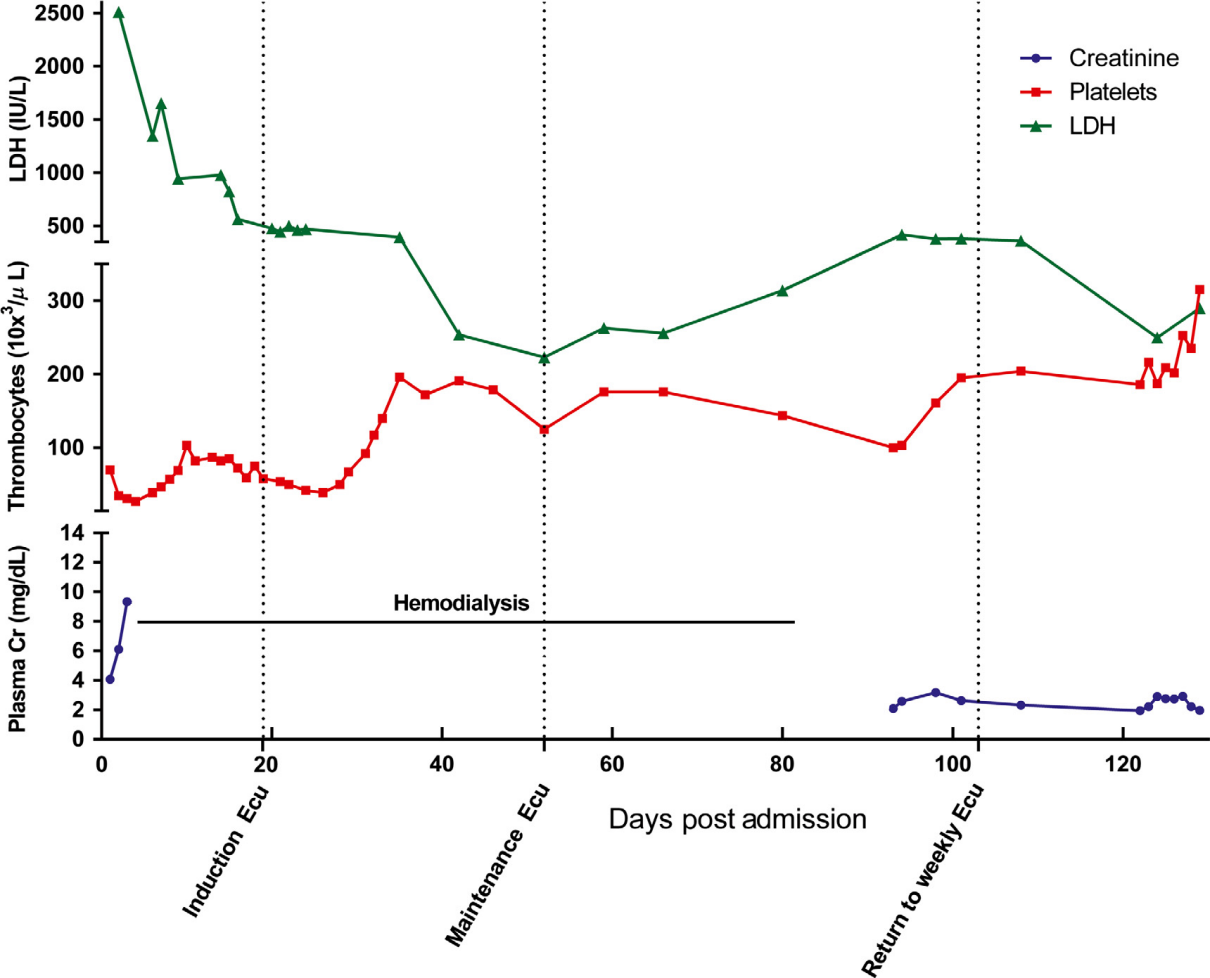
Laboratory findings associated with clinical course. Eculizumab induction with 900 mg IV weekly was started at day 19. Transition to maintenance dosing with 1200 mg IV every other week occurred after day 52. Due to decline in clinical status as evidenced by decreased platelets, rise in LDH and Cr, patient was transitioned back to weekly eculizumab dosing on day 103.

The patient was started on induction eculizumab at 900 mg IV weekly for 4 weeks and responded well with improvement in platelet count and renal function. He was transitioned to every‐other‐week maintenance eculizumab and hemodialysis was discontinued. However, approximately 6 weeks after switching to maintenance dosing, the patient developed recurrent abdominal pain, which he described as identical to his pain at initial presentation. Coincident with this, his platelet count declined, his LDH increased, and his renal function worsened (Fig. 1). These and other parameters, including haptoglobin, eGFR, and urinalysis, which were regularly monitored for response, demonstrated clinical decline. Due to these signs and symptoms, an increase was made to the dosing regimen of eculizumab rather than the addition of plasma therapy, which had proven ineffective for the patient at the time of diagnosis. He was transitioned back to weekly dosing of 900 mg IV, which once again led to improvement in symptoms and organ function.

The patient has remained clinically stable for 6 months on weekly dosing of 900 mg IV at the time of writing of this article. He has not experienced side effects from therapy such as headache, hypertension, diarrhea, or leukopenia, as are most commonly experienced 13; nor has he demonstrated signs or symptoms of meningitis in spite of the increased dosing regimen over this time period.

## Discussion

It is imperative to note that approximately 50% of patients with aHUS present with normal complement levels and therefore, normal levels cannot be used to exclude the diagnosis 3. Workup for aHUS should include functional assessment of complement via the total hemolytic complement (THC or CH50), which may be significantly low due to a genetic deficiency of one or more complement proteins. Genetic assessment can also be utilized for diagnostic purposes, however, diagnosis of aHUS can still be made in spite of normal genetic testing because all contributing genetic factors are not yet known.

Therapy with eculizumab has been associated with an increased risk of meningococcal infections (and other encapsulated organisms) due to the inhibitory effect on terminal complement. Vaccination against *N. Meningitidis* preferentially occurs 2 weeks prior to eculizumab initiation; however, given our patient's clinical acuity, therapy was started immediately after vaccine administration given that the risk of treatment delay outweighed the risk of developing a meningococcal infection. Prophylactic antibiotics against *N. Meningitidis* were administered for 2 weeks. With regard to duration of therapy, most patients with aHUS are treated with eculizumab indefinitely, however, some patients are able to be successfully weaned off treatment 14. One retrospective series reports a median exposure of 3 years 15.

Eculizumab administration by intravenous route requires that the medication be permitted to achieve room temperature prior to administration. It is infused over 35 min in adults, and 1–4 h in pediatric patients. It is not to be administered as a bolus injection or IV push. A maximum duration of 2 h of infusion in adults should not be exceeded. Should infusion reactions occur, infusion rates should be decreased or discontinued. Patients should be monitored for signs and symptoms of infusion reaction for at least 1 h after the completion of an infusion. Adequate supportive therapy is indicated throughout treatment, including maintenance of fluid balance, adequate blood pressure control, and electrolyte monitoring and replacement 13.

Due to the low prevalence of aHUS, the recommended dosing of eculizumab was based largely on the experience in treating PNH. The number of patients tested in the initial treatment that led to FDA approval was 87 16. For this reason, the standard dosing strategy may not be optimal for all patients as seen in the case presented. Examples of under‐dosing exist in the PNH literature also, where patients required dose adjustment due to lack of response 17, 18. To date, there are no reports in the literature that describe the need for dose titration of eculizumab in aHUS, though other cases of apparent under‐dosing have been observed during the transition to maintenance therapy (personal communication).

Several markers have been investigated that may help to evaluate efficacy of eculizumab, though none are yet proven to be reliable for dose‐modification. This remains best directed by clinical evaluation of the patient, as in our case. Potential markers that have been described include urine C5a and sC5b‐9, which decreased in studies immediately after first dose of eculizumab therapy 19. However, a significant fraction of patients with aHUS have been found to have normal circulating levels of C3, sC5b‐9, and c5A in serum, making these markers less clinically reliable 20. Other markers that decreased upon treatment include thrombomodulin (TM), a cell surface glycoprotein which is suggestive of decreased endothelial damage 19 and F1 + 2 (a prothrombin fragment) as well as D‐Dimer, indicating that eculizumab‐induced terminal complement blockade downregulates the coagulation cascade which may be useful for monitoring 21.

CH50 assays to monitor patient response in those treated with eculizumab have been proposed in the setting of both PNH and aHUS 22, 23. However, other studies have demonstrated CH50 to poorly correlate with patient response, and those authors propose following endothelial complement deposition assays 20. A contributor to the challenge in identifying a biomarker of response in aHUS patients is that complement dysregulation continues even in treated patients via chronic activation of the alternative complement pathway 21. Thus, eculizumab, at therapeutic levels, can reduce ongoing endothelial damage and renal injury from terminal complement in spite of ongoing chronic alternative complement pathway activation.

Both CH50 and circulating free eculizumab levels are the most promising markers of response in patients treated with eculizumab 22 and one group has suggested individualized dosing schedules based on eculizumab levels 24, but this strategy requires further validation.

## Conclusions

There are currently no clinically validated assays available that help to guide dose adjustment of eculizumab in patients with aHUS and instead, decisions must be made based on ongoing clinical evaluation of the patient. As demonstrated in the case of our patient, it may be necessary to increase the dose of eculizumab if clinical targets are not being met or initial response is lost after transition to maintenance schedule. Further pharmacodynamic studies of eculizumab, as well as the development of a dependable biomarker assay are needed to elucidate routine methods for determining necessary dose adjustments in patients with aHUS who experience inadequate control of their disease.

## Conflict of Interest

None declared.
